# Anti-CD38 Therapy With Daratumumab in High-Risk IgA Nephropathy

**DOI:** 10.1016/j.ekir.2026.106518

**Published:** 2026-04-06

**Authors:** Margot Poux, An S. De Vriese, Camille Cohen, Jean-Paul Duong Van Huyen, Eric Daugas, Anne Jolivot, Nizar Joher, Marie Matignon, Alexandre Hertig, Khalil El Karoui

**Affiliations:** 1 Nephrology Department, Sorbonne University, APHP, Hôpital Tenon, Paris, France; 2 Division of Nephrology and Infectious Disease, AZ Sint-Jan, Brugge, Belgium; 3 Department of Internal Medicine, Ghent University, Ghent, Belgium; 4 Nephrology Department, Paris Cité University, APHP, Hôpital Bichat, Paris, France; 5 Pathology Department, Paris Cité University, APHP, Hôpital Necker, Paris, France; 6 Nephrology Department, Hospices Civils de Lyon, Hôpital Edouard Herriot, Lyon, France; 7 Nephrology and transplantation Department, Paris Est University, APHP, Hôpital Henri Mondor, Créteil, France; 8 Nephrology Department, Hôpital Foch, Suresnes, France

## Introduction

Patients with IgA nephropathy (IgAN) are at high risk of progressive kidney disease, particularly in cases of persistent proteinuria, reduced estimated glomerular filtration rate (eGFR), or severe histological lesions.^1^ Current clinical trials in IgAN are investigating treatments aimed at modulating IgA production and B-cell or plasma cell activation, as well as attenuating glomerular inflammation and its downstream effects.^2^

Among these approaches, anti-CD38 therapy—targeting plasma cells—has emerged as a promising option to improve key biomarkers of IgAN progression, including circulating IgA levels, proteinuria, and eGFR, as recently demonstrated with felzartamab (which acts via antibody-dependent cellular cytotoxicity) in the phase 2 IGNAZ study.^3^ Notably, another anti-CD38 monoclonal antibody, daratumumab (which acts preferentially via complement-dependent cytotoxicity), has been used for several years in plasma cell disorders such as multiple myeloma and amyloid light-chain amyloidosis, with robust efficacy and a favorable safety profile. Although never formally investigated to date, daratumumab may represent a promising option for high-risk IgAN.

In the present study, we characterize the effects of daratumumab in 4 patients with IgAN with different presentations (including IgA vasculitis and IgAN associated with inflammatory bowel disease) and severe disease.

## Case Presentation

### Patients With IgAN Treated With Daratumumab

Four patients with IgAN treated with daratumumab were retrospectively identified from 4 tertiary care centers in France and Belgium. Two patients (1 and 3) initially presented with monotypic IgA deposits suggestive of monoclonal gammopathy of renal significance. However, because no plasma cell clone was eventually identified despite extensive investigations, including light-chain RNA sequencing, these cases were reclassified as IgAN. Indeed, patient 1, who initially had IgA lambda–predominant deposits on native kidney biopsy, demonstrated mesangial polytypic IgA deposits on repeat biopsy at the time of posttransplant recurrence, confirming the diagnosis of polytypic IgAN. The other patients (2 and 4) received daratumumab as compassionate-use treatment in the absence of other therapeutic options for progressive IgAN.

**Figure 1:**
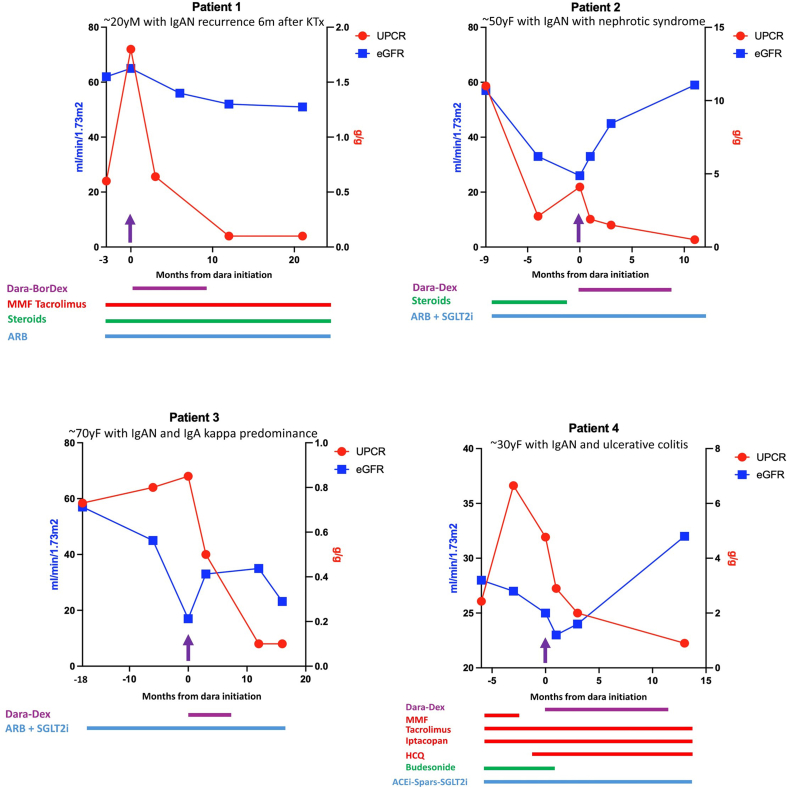
Evolution of eGFR and proteinuria before and after treatment with daratumumab in patients 1–4. ARB, angiotensin receptor blocker; Bor, bortezomib; Dara, daratumumab; Dex, dexamethasone; eGFR, estimated glomerular filtration rate; F, female; IgAN, IgA nephropathy; KTx, kidney transplant; m, months; M, male; MMF, mycophenolate; HCQ, hydroxychloroquine; SGLT2i, sodium-glucose cotransporter 2 inhibitor; Spars, sparsentan; UPCR, urinary protein-to-creatinine ratio; y, year.

Patient characteristics are presented in Figure 1 and Supplementary Table S1. The median age was 42 years, median eGFR was 26 ml/min per 1.73 m^2^, median serum albumin was 32.8 g/l, and median urinary protein-to-creatinine ratio was 2.7 g/g despite maximization of nephroprotective therapies. Persistent microscopic hematuria was present in all patients. The median delay from IgAN diagnosis was 32 months, and the last kidney biopsy was performed 2 to 36 months before daratumumab initiation. All but 1 patient (patient 3) had previously received various immunosuppressive drugs, including systemic steroids, budesonide, mycophenolate mofetil, iptacopan, tacrolimus, or hydroxychloroquine. Daratumumab (1800 mg subcutaneous injections, weekly for 1–2 months, then biweekly for 2–5 months, then monthly) plus bortezomib and dexamethasone in patient 1 and plus dexamethasone only in patients 2 and 4 was given for 9 to 12 months. After a median follow-up of 13.5 months, all patients presented with a favorable evolution (stable or increased eGFR, proteinuria reduction of 30%–90%, and complete resolution of hematuria), with a median eGFR of 38.5 ml/min per 1.73 m^2^, median serum albumin of 42 g/l, and median urinary protein-to-creatinine ratio of 0.3 g/g. Daratumumab allowed discontinuation or dose reduction of other immunosuppressive medications. Treatment was associated with complete resolution of inflammatory lesions (despite persistent mesangial IgA deposits) in 2 patients who underwent repeat renal biopsy (patients 1 and 2, Supplementary Table S1, Supplementary Figure S1). The beneficial effects were sustained for 10 to 12 months after the last injection in 2 evaluable patients. Safety was acceptable, with 1 episode of pneumonia (patient 1), which resolved after 7 days of antibiotic therapy. No significant cytopenia or hypogammaglobulinemia was observed.

### Circulating IgA Levels in Patients With CKD Treated With Daratumumab

We quantified IgA, IgG, and IgM levels in prospective serum samples from the Dardar study (NCT04204980), evaluating desensitization with daratumumab in dialysis patients.^4^ Treatment with daratumumab led to a marked reduction in serum IgA levels in patients with CKD, whereas IgG and IgM levels showed only a transient, nonsignificant decline (Supplementary Figure S2).

## Discussion

Treatment with daratumumab, a humanized anti-CD38 monoclonal antibody routinely used in plasma cell dyscrasias, was well tolerated and led to favorable clinical outcomes in 4 patients with high-risk IgAN (Table 1).

**Table 1:** Teaching points

Targeting CD38 has shown promising results in IgAN in a phase 2 study in patients with persistent proteinuria.
The anti-CD38 monoclonal antibody, daratumumab, is routinely used in multiple myeloma and amyloidosis and has a favorable safety profile.
Daratumumab appears a valuable therapeutic option in patients with severe IgAN, although further studies are warranted to confirm its long-term efficacy and safety.

IgAN, IgA nephropathy.

All patients had severe IgAN characterized by persistent proteinuria and/or progressive renal dysfunction despite optimized renin-angiotensin system blockade and/or corticosteroid therapy. Daratumumab, administered for varying durations, produced meaningful clinical improvement in all cases, with stabilization or recovery of renal function and reduction of proteinuria of up to 90% from baseline. Hematuria, a marker of IgAN activity, resolved in all patients. Importantly, repeat biopsies performed 3 and 4 months after daratumumab initiation revealed commensurate histological resolution of inflammatory lesions, although IgA deposits remained detectable. The beneficial effects of daratumumab persisted for several months after treatment discontinuation. Treatment was generally well tolerated in this series, with only 1 serious infectious complication occurring in a kidney transplant recipient who was concurrently receiving additional immunosuppressants. No patient had to discontinue daratumumab because of adverse effects.

The safety and efficacy outcomes observed with daratumumab in our case series compare favorably with those reported for felzartamab, another anti-CD38 monoclonal antibody currently under investigation in IgAN.^3^ Notably, patients included in the IGNAZ trial generally had less severe disease; however, the reduction in proteinuria and preservation of kidney function achieved with daratumumab were at least as impressive. Subcutaneous administration of daratumumab was favored in all patients because of better tolerability and comparable efficacy.^3^^,^^5^

Daratumumab has become a core component of standard-of-care regimens in multiple myeloma and amyloid light-chain amyloidosis. Off-label use of daratumumab in other severe autoimmune conditions, including lupus nephritis, antineutrophil cytoplasmic autoantibody–associated vasculitis, and membranous nephropathy, has shown encouraging results.^6^ Daratumumab is already commercially available, widely used in clinical practice, and familiar to physicians. In addition, it is expected to be more affordable than the anticipated cost of emerging anti-CD38 molecules.

Two patients presented with monotypic IgA deposits on kidney biopsy, prompting treatment with daratumumab despite the absence of a detectable circulating monoclonal component. This observation aligns with data from large IgAN cohorts, in which “monotypic” IgAN has been described without plasma cell dyscrasia.^7^ Specific approaches, such as use of heavy-chain or light-chain immunofluorescence, frequently unmask polytypic deposits in this context.^8^ Importantly, in our series, patients with “monotypic” and “polytypic” deposits demonstrated comparable clinical responses to anti-CD38 therapy.

Interestingly, in the Dardar study, a 2-month course of daratumumab led to a significant reduction in total circulating IgA levels, persisting for ≤1 year, consistent with previous observations in patients with monoclonal gammopathy of renal significance and underlying plasma cell clones.^5^ This sustained decrease in IgA levels may contribute, at least in part, to daratumumab’s efficacy.

This report has several limitations inherent to its retrospective and observational design. The small sample size and the heterogeneity of patient presentations preclude any definitive conclusions about the long-term efficacy and safety of daratumumab in IgAN. Concomitant therapies, such as dexamethasone, may have contributed to the observed outcomes. Lastly, total IgA levels may not correlate with galactose-deficient-IgA1 levels in the Dardar study.

In summary, daratumumab, a routinely available anti-CD38 monoclonal antibody with a favorable safety profile, may offer therapeutic benefit in selected patients with severe IgAN. Considering that prospective, controlled studies confirm its long-term efficacy and safety in this population, daratumumab may be an attractive addition to the therapeutic armamentarium in IgAN.

## Disclosure

All the authors declared no competing interests.

## Patient Consent

The authors declare that they have obtained consent from the patients discussed in the report.

## Data Availability Statement

The data in this study were extracted from confidential medical records. European legislation does not authorize their free use and transfer. These data cannot be made freely available. Data may be shared as part of a collaborative project, subject to further validation by institutional review board and patient consent.

## Supplementary Material

Supplementary File (PDF) **Detailed Patient Description. Circulating IgA Levels in Patients With CKD Treated With Daratumumab. Figure S1.** Representative images of kidney biopsies performed in patient 2 before and after daratumumab. **Figure S2.** Evolution of IgG, IgM, and IgA circulating levels in 13 patients with end-stage kidney disease treated with i.v. daratumumab 16 mg/kg for 8 weekly injections. **Table S1.** Detailed clinical and biological characteristics before and after daratumumab. STROBE Checklist.

## Footnotes

Supplementary File (PDF)

**Detailed Patient Description.**

**Circulating IgA Levels in Patients With CKD Treated With Daratumumab.**
**Figure S1.** Representative images of kidney biopsies performed in patient 2 before and after daratumumab. **Figure S2.** Evolution of IgG, IgM, and IgA circulating levels in 13 patients with end-stage kidney disease treated with i.v. daratumumab 16 mg/kg for 8 weekly injections. **Table S1.** Detailed clinical and biological characteristics before and after daratumumab. STROBE Checklist.
