# Supplementary material for: Anti-CD38 Therapy With Daratumumab in High-Risk IgA Nephropathy
Source: Kidney Int Rep. 2026 Apr 6;11(7):106518. doi: 10.1016/j.ekir.2026.106518 (PMC13206733; doi:10.1016/j.ekir.2026.106518)
Supplement: Supplementary File (PDF) — Detailed Patient Description. Circulating IgA Levels in Patients With CKD Treated With Daratumumab. Figure S1. Representative images of kidney biopsies performed in patient 2 before and after daratumumab. Figure S2. Evolution of IgG, IgM, and IgA circulating levels in 13 patients with end-stage kidney disease treated with i.v. daratumumab 16 mg/kg for 8 weekly injections. Table S1. Detailed clinical and biological characteristics before and after daratumumab. STROBE Checklist. [file mmc1.pdf]

Supplementary material

Detailed patient description

Patient 1 had a history of rapidly progressive glomerulonephritis with extracapillary proliferation and monotypic IgA lambda deposits. End-stage renal disease developed despite treatment with steroids, plasma exchange, and a combination of bortezomib, cyclophosphamide, and dexamethasone. Extensive immuno-hematological investigations—including ANCA, anti-GBM antibodies, serum and urine immunofixation, serum free light chains, bone marrow cytology and biopsy, and peripheral clonality studies—were unremarkable. The patient underwent kidney transplantation two years later. Six months post-transplant, an endocapillary and extracapillary proliferative glomerulonephritis with polytypic mesangial IgA deposits developed (IFTA 10%, globally sclerosed glomeruli 0%), associated with kidney dysfunction and proteinuria (**Figure 1**). Daratumumab (1800 mg subcutaneous injections, weekly for 2 months, bimonthly for 2 months, then monthly, associated with DXM 40mg) was initiated in combination with bortezomib for a total duration of 9 months, resulting in stabilization of renal function and disappearance of proteinuria and hematuria. A repeat kidney biopsy 3 months after treatment initiation showed resolution of inflammatory lesions, although polytypic mesangial IgA deposits persisted (IFTA 5%, globally sclerosed glomeruli 0%). A pulmonary infection (needing a 7-day hospitalization) diagnosed 8 months into treatment was successfully managed with antibiotics (7 days) despite no bacteria identification. At last follow-up, gammaglobulins were 8.7g/l.

Patient 2 presented with nephrotic syndrome and rapidly progressive renal function loss due to a diffuse proliferative IgAN (eGFR 57 ml/min/1.73m², serum albumin 23 g/L, protein-to-creatinine ratio [PCR] 8 g/g, MEST-C: M1 E1 S1 T0 C2, **Figure 1-S1**). Despite a 6-month course of high dose corticosteroids (3x 500-mg IV injections, followed by prednisone at 1 mg/kg for one month, with progressive tapering over six months), proteinuria and renal dysfunction persisted. A second kidney biopsy, performed 9 months after steroid initiation, revealed ongoing diffuse proliferation (eGFR 26 ml/min/1.73m², PCR 4 g/g, MEST-C: M1 E1 S1 T1 C1, IFT 10%, globally sclerosed glomeruli 60% **Figure 1-S1**). The patient declined cyclophosphamide therapy. Given the severity of the disease and steroid resistance, daratumumab was initiated on a compassionate-use basis (1800 mg subcutaneous injections, weekly for 2 months, bimonthly for 2 months, then monthly for a total of 9 months, associated with DXM 20mg). A third biopsy 4 months after starting daratumumab showed complete resolution of inflammatory lesions with persistent mesangial IgA deposits (MEST-C: M1 E0 S1 T1 C0, **Figure S1,** IFTA 40%, globally sclerosed glomeruli 50%). At 11-month follow-up, renal function had markedly improved (eGFR from 26 to 59 ml/min/1.73m², confirmed in repeat eGFR), proteinuria had

decreased by 87%, and hematuria had resolved. No infectious complications occurred. No hypogammaglobulinemia was reported.

Patient 3 had a childhood history of IgA vasculitis with persistent hematuria and recurrent episcleritis. Later, worsening proteinuria and renal dysfunction led to a diagnosis of inflammatory IgAN with predominant IgA kappa deposits (eGFR 28ml/min/1,73m^2, proteinuria 0,73g/d, MEST-C: M1 E0 S1 T0 C1). Immuno-hematological workup—including serum and urine immunofixation, serum free light chains, bone marrow cytology, and bone marrow light chain mRNA sequencing—was unremarkable. Despite optimal nephroprotection for ~3 years, proteinuria persisted and renal function declined (**Figure 1**). Daratumumab monotherapy was initiated (1800 mg subcutaneous injections, weekly for 2 months, bimonthly for 2 months, then monthly for a total of 6 months, without DXM), resulting in improvement of renal function (eGFR from 17 to 23 ml/min/1.73m^2) and resolution of proteinuria and hematuria. Treatment was well tolerated, and no hypogammaglobulinemia was noticed.

Patient 4 had a history of severe ulcerative colitis, previously treated with budesonide (entocort), azathioprine, and anti-TNFα therapy. She developed severe nephrotic syndrome and progressive decline in kidney function due to a diffuse endocapillary and extracapillary proliferative glomerulonephritis. Despite aggressive immunosuppression, including pulse steroids (pulse steroids and methylprednisolone tapered 1 year before daratumumab), mycophenolate, tacrolimus, iptacopan, and hydroxychloroquine, as well as treatment with an SGLT2i and sparsentan, proteinuria and renal dysfunction (eGFR 25 ml/min/1.73m^2, PCR 4.5 g/g, **Figure 1**) progressed. Treatment with daratumumab (1800 mg subcutaneous injections, weekly for 1 month, bimonthly for 5 months, then monthly, associated with DXM 20mg) for 12 months led to stabilization of renal function and resolution of proteinuria and hematuria, even with dose reduction or discontinuation of concomitant immunosuppressants. No infectious complications nor hypogammaglobulinemia occurred during treatment.

Circulating IgA levels in CKD patients treated with daratumumab

In an additional pathophysiological approach, we leveraged the Dardar study to prospectively assess circulating IgA levels in a non-oncological CKD context (eg dialysis patients waiting for kidney transplantation). The Dardar study (NCT04204980) is a phase 2, monocentric open-label study conducted to evaluate safety and efficacy of desensitization with daratumumab (8 weekly infusions of 16 mg/kg) in dialysis patients waiting for kidney transplantation.

To evaluate the effect of daratumumab on circulating IgA levels of CKD patients, we analyzed prospective serum samples from the Dardar Study and quantified IgA, IgG and IgM levels by immunonephelometry (**Figure S2**).

13 hemodialysis patients were included in the phase 2 Dardar study. Patient characteristics have been previously described (4). The causes of ESRD include lupus nephritis (n=2), or unknown etiology (n=11). At baseline, IgA levels were 2.39g/l (SD 1,40).

After 8 weekly intravenous infusions of daratumumab 16mg/kg, mean IgA levels were 0.8 (SD 0.42) at month 3, 1.0 (SD 0.65) at month 6 and 1.38 (SD 0.79) at month 12, corresponding to a mean reduction from baseline of 66%, 57% and 42%, respectively (Supplemental Figure 1). The reduction of IgG levels was 39,2%, 34.4% and 16.5% at month 3, month 6 and month 12, respectively. The reduction of IgM levels was 40.8%, 31.7% and 6.5% at month 3, month 6 and month 12, respectively. At month 12, mean IgG level was 10.9 g/l and mean IgM level was 0.6 g/l.

	Presentation at dara initiation							Last follow-up from dara initiation						
**Medical History**	**eGFR**	**Alb**	**UPCR**	**UHem**	**Last IgAN KB (delay/dara initiation)**	**TTT**	**Protocol**	**FU**	**eGFR**	**Alb**	**UPCR**	**UHem**	**TTT**	**Repeat KB (delay/dara initiation)**
P1; M, ~20y; IgAN recurrence 6m after KTx	58	NA	1.5	100000	M0E1S0T0C1 (3m)	steroids, MMF, tacro	Dara Bor Dex, 9m	21m	51	48	0.1	1000	MMF, tacro pred, ARB	M1E1S0T0 C0 (3 m)
P2; F, ~50y, IgAN associated nephrotic syndrome (M1E1S1T0C2) treated with 6m steroids	26	32.8	4.1	15000	M1E1S1T1 C1 (2m)	steroids, ARB, SGLT2i	Dara Dex, 9m	11m	59	42	0.5	<1000	ARB, SGLT2i	M1E0S1T1 C0 (4m)
P3; F, ~70y, childhood IgA vasculitis, persistent hematuria; IgAN with IgA kappa predominance, no detected clone	17	36	0.7	100000	M1E0S1T0 C1 (35m)	ACEi	Dara Dex, 6m	16m	23,2	NA	0.1	4000	ACEi	NA
P4; F, ~30y, Severe ulcerative colitis; Diffuse endocapillary and crescentic IgAN, resistant to steroids, MMF, tacro; then ipta, budesonide, sparsentan	25	27	4.4	90000	M1E1S1T0 C1 (29m)	ipta, tacro, HXQ, SGLT2i, ACEi, sparsentan	Dara Dex, 12m	13m	32	42	0.9	7000	low dose tacro, HXQ, SGLT2i, sparsent an	NA

Supplemental Table 1. Detailed clinical and biological characteristics before and after daratumumab.

Alb: serum albumin (g/l); Dara: daratumumab; eGFR: estimated glomerular filtration rate (ml/mn/1,73m^2); FU: follow-up (months); HXQ: hydroxychloroquine; UHem: hematuria evaluated by urine microscopy analysis and flow cytometry (/ml, N<10000/ml); ipta: iptacopan; MMF: mycophenolate mofetil ; UPCR: urinary protein/creatinine ratio in spot collection, g/g; Tacro: tacrolimus; TTT: treatment

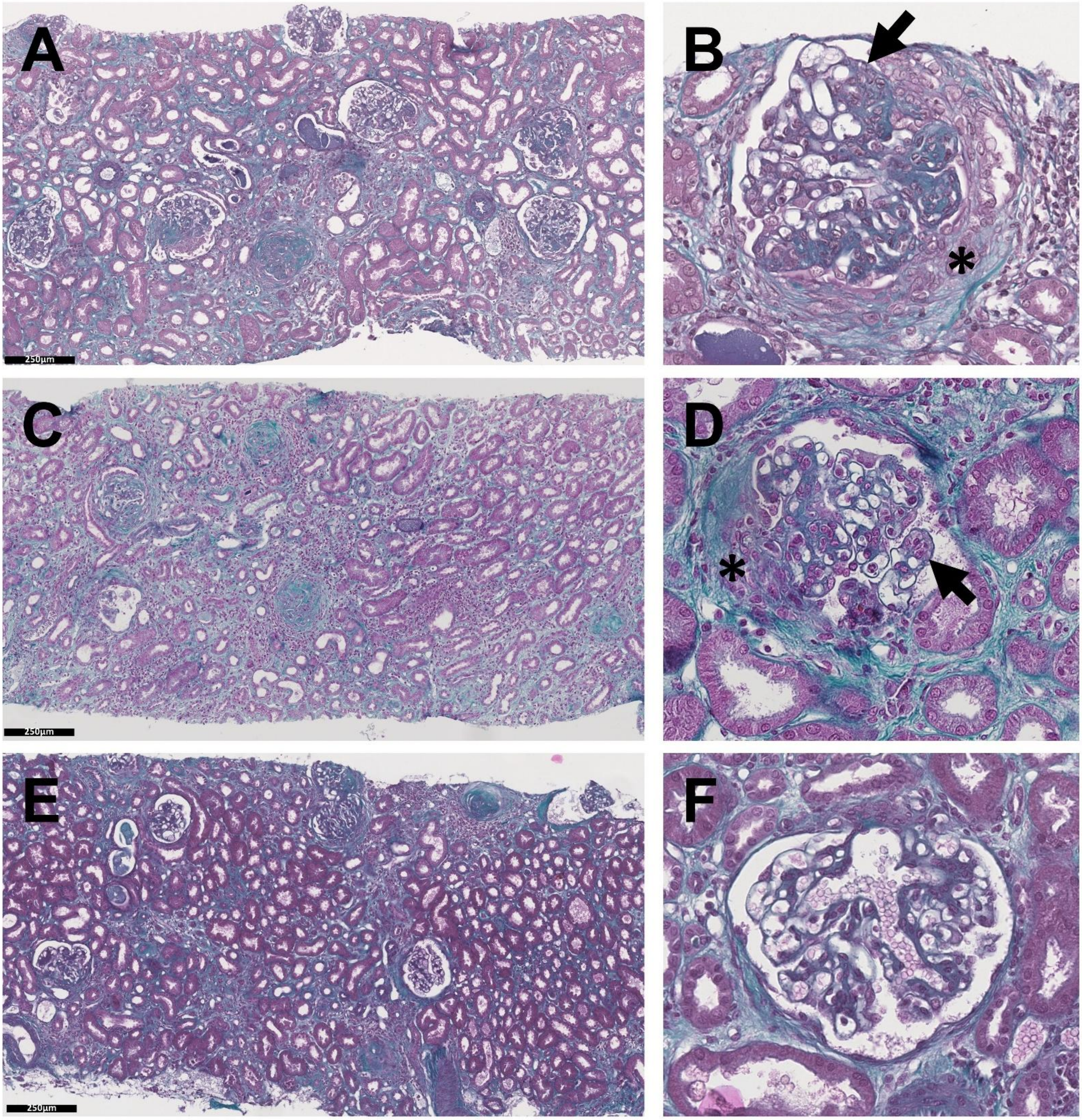

Supplemental Figure 1

Representative images of kidney biopsies performed in patient 2 before and after daratumumab

A-B First kidney biopsy. Note endocapillary (arrow) and extracapillary (star) proliferation. **C-D** Second kidney biopsy performed after steroid treatment. Persistence of endocapillary (arrow) and extracapillary (star) proliferation. **E-F** Third kidney biopsy 4 months after daratumumab initiation showing resolution of glomerular inflammatory lesions. Masson trichrome, original magnification x100, x400

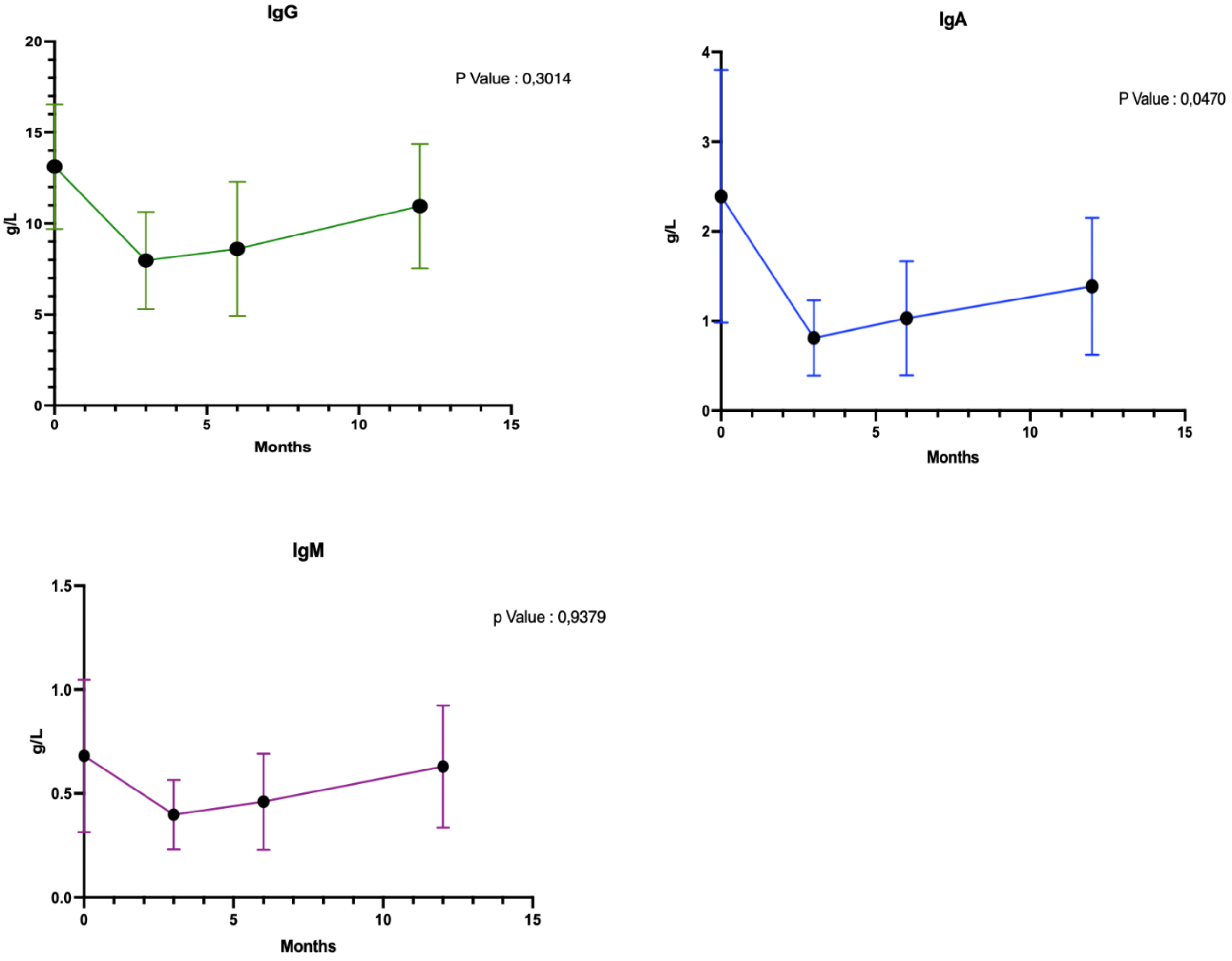

Supplemental Figure 2 Evolution of IgG, IgM, and IgA circulating levels in 13 ESKD patients treated with intravenous daratumumab 16mg/kg for 8 weekly injections.

STROBE Statement—checklist of items that should be included in reports of observational studies

	Item No.	Recommendation	Page No.	Relevant text from manuscript
**Title and abstract**	1	(*a*) Indicate the study's design with a commonly used term in the title or the abstract	1	
		(*b*) Provide in the abstract an informative and balanced summary of what was done and what was found	1	
**Introduction**				
Background/rationale	2	Explain the scientific background and rationale for the investigation being reported	3	
Objectives	3	State specific objectives, including any prespecified hypotheses	3	
**Methods**				
Study design	4	Present key elements of study design early in the paper	3	
Setting	5	Describe the setting, locations, and relevant dates, including periods of recruitment, exposure, follow-up, and data collection	3	
Participants	6	(*a*) *Cohort study*—Give the eligibility criteria, and the sources and methods of selection of participants. Describe methods of follow-up<br>*Case-control study*—Give the eligibility criteria, and the sources and methods of case ascertainment and control selection. Give the rationale for the choice of cases and controls<br>*Cross-sectional study*—Give the eligibility criteria, and the sources and methods of selection of participants	4	
		(*b*) *Cohort study*—For matched studies, give matching criteria and number of exposed and unexposed<br>*Case-control study*—For matched studies, give matching criteria and the number of controls per case		

Variables	7	Clearly define all outcomes, exposures, predictors, potential confounders, and effect modifiers. Give diagnostic criteria, if applicable	4
Data sources/ measurement	8*	For each variable of interest, give sources of data and details of methods of assessment (measurement). Describe comparability of assessment methods if there is more than one group	*4*
Bias	9	Describe any efforts to address potential sources of bias	NA
Study size	10	Explain how the study size was arrived at	NA

Continued on next page

Quantitative variables	11	Explain how quantitative variables were handled in the analyses. If applicable, describe which groupings were chosen and why	
Statistical methods	12	(*a*) Describe all statistical methods, including those used to control for confounding	3
		(*b*) Describe any methods used to examine subgroups and interactions	NA
		(*c*) Explain how missing data were addressed	NA
		(*d*) *Cohort study*—If applicable, explain how loss to follow-up was addressed<br>*Case-control study*—If applicable, explain how matching of cases and controls was addressed<br>*Cross-sectional study*—If applicable, describe analytical methods taking account of sampling strategy	NA
		(*e*) Describe any sensitivity analyses	
**Results**			
Participants	13*	(a) Report numbers of individuals at each stage of study—eg numbers potentially eligible, examined for eligibility, confirmed eligible, included in the study, completing follow-up, and analysed	4
		(b) Give reasons for non-participation at each stage	NA
		(c) Consider use of a flow diagram	NA
Descriptive data	14*	(a) Give characteristics of study participants (eg demographic, clinical, social) and information on exposures and potential confounders	4, Table 1
		(b) Indicate number of participants with missing data for each variable of interest	NA
		(c) *Cohort study*—Summarise follow-up time (eg, average and total amount)	NA
Outcome data	15*	*Cohort study*—Report numbers of outcome events or summary measures over time	*4*
		Case-control study—Report numbers in each exposure category, or summary measures of exposure	
		Cross-sectional study—Report numbers of outcome events or summary measures	

Main results	16	(*a*) Give unadjusted estimates and, if applicable, confounder-adjusted estimates and their precision (eg, 95% confidence interval). Make clear which confounders were adjusted for and why they were included	NA
		(*b*) Report category boundaries when continuous variables were categorized	NA
		(*c*) If relevant, consider translating estimates of relative risk into absolute risk for a meaningful time period	NA

Continued on next page

Other analyses	17	Report other analyses done—eg analyses of subgroups and interactions, and sensitivity analyses	NA
**Discussion**			
Key results	18	Summarise key results with reference to study objectives	5
Limitations	19	Discuss limitations of the study, taking into account sources of potential bias or imprecision. Discuss both direction and magnitude of any potential bias	5
Interpretation	20	Give a cautious overall interpretation of results considering objectives, limitations, multiplicity of analyses, results from similar studies, and other relevant evidence	5
Generalisability	21	Discuss the generalisability (external validity) of the study results	6
**Other information**			
Funding	22	Give the source of funding and the role of the funders for the present study and, if applicable, for the original study on which the present article is based	6

*Give information separately for cases and controls in case-control studies and, if applicable, for exposed and unexposed groups in cohort and cross-sectional studies.

Note: An Explanation and Elaboration article discusses each checklist item and gives methodological background and published examples of transparent reporting. The STROBE checklist is best used in conjunction with this article (freely available on the Web sites of PLoS Medicine at http://www.plosmedicine.org/, Annals of Internal Medicine at http://www.annals.org/, and Epidemiology at http://www.epidem.com/). Information on the STROBE Initiative is available at www.strobe-statement
